# Role of Hepatocyte Transporters in Drug-Induced Liver Injury (DILI)—In Vitro Testing

**DOI:** 10.3390/pharmaceutics15010029

**Published:** 2022-12-22

**Authors:** Péter Tátrai, Franciska Erdő, Péter Krajcsi

**Affiliations:** 1 SOLVO Biotechnology, Charles River Laboratories Hungary, H-1117 Budapest, Hungary; 2 Faculty of Information Technology and Bionics, Pázmány Péter Catholic University, H-1088 Budapest, Hungary; 3 Department of Morphology and Physiology, Faculty of Health Sciences, Semmelweis University, H-1081 Budapest, Hungary; 4 Habilitas Kft, H-1025 Budapest, Hungary

**Keywords:** hepatotoxicity, DILI, cholestasis, BSEP, transporters, in vitro ADME

## Abstract

Bile acids and bile salts (BA/BS) are substrates of both influx and efflux transporters on hepatocytes. Canalicular efflux transporters, such as BSEP and MRP2, are crucial for the removal of BA/BS to the bile. Basolateral influx transporters, such as NTCP, OATP1B1/1B3, and OSTα/β, cooperate with canalicular transporters in the transcellular vectorial flux of BA/BS from the sinusoids to the bile. The blockage of canalicular transporters not only impairs the bile flow but also causes the intracellular accumulation of BA/BS in hepatocytes that contributes to, or even triggers, liver injury. In the case of BA/BS overload, the efflux of these toxic substances back to the blood via MRP3, MRP4, and OST α/β is considered a relief function. FXR, a key regulator of defense against BA/BS toxicity suppresses de novo bile acid synthesis and bile acid uptake, and promotes bile acid removal via increased efflux. In drug development, the early testing of the inhibition of these transporters, BSEP in particular, is important to flag compounds that could potentially inflict drug-induced liver injury (DILI). In vitro test systems for efflux transporters employ membrane vesicles, whereas those for influx transporters employ whole cells. Additional in vitro pharmaceutical testing panels usually include cellular toxicity tests using hepatocytes, as well as assessments of the mitochondrial toxicity and accumulation of reactive oxygen species (ROS). Primary hepatocytes are the cells of choice for toxicity testing, with HepaRG cells emerging as an alternative. Inhibition of the FXR function is also included in some testing panels. The molecular weight and hydrophobicity of the drug, as well as the steady-state total plasma levels, may positively correlate with the DILI potential. Depending on the phase of drug development, the physicochemical properties, dosing, and cut-off values of BSEP IC_50_ ≤ 25–50 µM or total C_ss,plasma_/BSEP IC_50_ ≥ 0.1 may be an indication for further testing to minimize the risk of DILI liability.

## 1. Introduction

Membrane transporters comprise two large superfamilies, the solute carrier (SLC) superfamily and the ATP-binding cassette (ABC) transporter superfamily. The SLC superfamily typically facilitates the influx of substrates, but as most SLCs are exchangers, they may also operate as efflux transporters [1]. Mechanistically, ABC transporters are simpler, with most of them being primary active transporters that fulfil an efflux function.

Some transporters are better known for their roles in mediating the absorption–distribution–metabolism–excretion (ADME) of xenobiotics, including drugs [2]. However, even these transporters have important physiological substrates whose homeostasis may be impaired in the case of transporter inhibition. Such physiological substrates, if their plasma levels change in parallel with those of the substrate drugs, may serve as biomarkers and surrogate probes in drug–drug interaction studies. This aspect has been extensively reviewed elsewhere [3,4,5] and will not be covered in this review.

Some hepatocytic transporters, on the other hand, have mostly been investigated for their roles in the homeostasis of a special class of physiological substrates: bile acids and bile salts. The impaired homeostasis of bile components—bilirubin being another classical example [6]—may lead to toxicity or other adverse events, and elevated plasma levels of such molecules may indicate the toxic potential of drugs. The homeostasis of physiological substrates is a complex process involving the extensive interplay of transporters and enzymes. Elimination through the liver is mediated by basolateral influx transporters and apical efflux transporters, whereas reabsorption occurs via enterohepatic or cholehepatic shunts. A third transport pathway provided by basolateral efflux functions is a relief pathway that protects hepatocytes from the intracellular accumulation of physiological substrates to tissue-toxic levels and ultimately redirects these substrates toward urinary excretion.

## 2. Cholestasis—A Major Form of Drug-Induced Liver Injury

Drug-induced liver injury (DILI) is the leading cause of drug development termination and drug withdrawal [7,8]. The package inserts of many marketed drugs display black box warnings about the risk of hepatic injury. Drugs may cause liver injury shortly after administration in a dose-dependent manner that is labelled as “intrinsic” hepatotoxicity. Idiosyncratic hepatotoxicity is more challenging as it may occur weeks or months after treatment [9]. Idiosyncratic DILI was traditionally considered dose-independent, but this concept has been challenged lately [10]. Mechanistically, a drug may cause cholestatic, hepatocellular, or mixed liver injury [11,12]. Cholestatic and mixed cases constitute 20–40% and 12–20% of DILI cases, respectively [13]. Based on these and additional clinical data, different scoring systems of DILI have been proposed [14].

Cholestasis is defined as decreased bile flow. Depending on the cause and site of the disturbance, cholestasis may be classified as intrahepatic if the dysfunction (e.g., inflammation) is intrinsic to the liver or extrahepatic if the extrahepatic portion of the biliary tree is obstructed. At the level of clinical laboratory findings, according to the Council for International Organizations of Medical Sciences (CIOMS) criteria, liver injury is designated as cholestatic if (i) serum alkaline phosphatase (AP) activity is elevated to >2-fold of the upper limit of normal, or (ii) the alanine amino transferase (ALT) serum activity to AP serum activity ratio (ALT/AP) is ≤2 [11]. The increased release of AP into the serum was shown to be induced by elevated intracellular bile salt levels characteristic of cholestasis [15]. Cholestasis has long been associated with defects of the transporter function as inherited forms of cholestasis, referred to as various types of progressive familial intrahepatic cholestasis (PFIC), are linked with mutations in transporter-coding genes. The PFIC type 1 is linked with mutations in *ATP8B1*, the PFIC type 2 with mutations in *ABCB11*/BSEP, and the PFIC type 3 with mutations in *ABCB4*/MDR3. Interestingly, children with different types of PFIC display a different spectrum of symptoms: failure to thrive is common in PFIC1, elevated ALT is common in PFIC2 (BSEP deficiency), and thrombocytopenia is common in PFIC3 (MDR3 deficiency) [16].

### 2.1. Bile Salt Transporters

Bile acids (BA) synthesized de novo in the liver undergo amidation to form bile salts (BS), also called monovalent bile acids. Most bile acids are then conjugated with glycine in healthy humans and taurine in rodents. Both BA and BS can undergo Phase II metabolism to yield sulfated or glucuronidated BA/BS. The Phase II conjugates of monovalent BS are also called divalent bile salts. The transport of BA/BS into hepatocytes is mediated by the sinusoidal influx transporters sodium taurocholate co-transporting polypeptide (NTCP)/solute carrier 10A1 (*SLC10A1*), organic anion transporting polypeptide 1B1 (OATP1B1)/solute carrier organic anion transporter 1B1 (*SLCO1B1*), and OATP1B3/*SLCO1B3*. Canalicular transport is mainly mediated using a bile salt export pump (BSEP)/*ABCB11* and multidrug resistance-associated protein 2 (MRP2)/*ABCC2*. MRP3/*ABCC3* and MRP4/*ABCC4* provide the sinusoidal efflux function. The heterodimeric organic solute transporter (OSTα/β)/*SLC51A/B* is a facilitative transporter and may mediate transport into as well as out of the hepatocyte [17].

#### 2.1.1. NTCP

NTCP is one of the several membrane transporters highly expressed on the sinusoidal (basolateral) membrane of hepatocytes [18]. NTCP is the primary influx transporter for the sodium-dependent BS uptake from portal blood [19,20]. The cloning of human NTCP [21] greatly facilitated the identification of its substrates and inhibitors [22,23,24,25,26,27].

The significance of NTCP was clearly demonstrated in a study on knockout mice that displayed hypercholanemia mainly composed of conjugated BS. Several variants described in humans [28,29] showed the impaired transport of bile salts in vitro. The leading clinical phenotype was the elevation of the plasma BS levels that could be mild even in homozygotes [30] and compound heterozygotes [31]. Heterozygotes for the c.800C > T (p.S267F) allele showed no alteration in ursodeoxycholic acid (UDCA) and glycoursodeoxycholic acid (GUDCA) pharmacokinetics after UDCA dosing [32]. This variant displayed about an eight-fold decreased clearance in vitro, resulting from an eight-fold increase in K_m_ and an unchanged V_max_ for taurocholate [29].

A case report described three children with hypercholanemia persisting beyond 1 year who were shown to be homozygotes for this mutation [33]. The non-synonymous c.755G > A polymorphism (p.R252H) resulted in extremely elevated plasma BS levels, but no cholestasis, jaundice, or liver dysfunction were observed [34,35]. This mild phenotype may be due to a shift in the BS profile towards more soluble and less hepatotoxic sulfated BS which was observed in both *SLC10A1* knockout mice and p.S267F humans [36].

In vitro drug inhibition studies have almost exclusively used taurocholate (TC) as the probe substrate, and the NTCP variants show striking differences in the transport of taurocholate and rosuvastatin [28,29]. In healthy humans, the plasma levels of TC are much lower relative to glycochenodeoxycholate (GCDC) [37], whereas in cholestasis, the TC levels may even exceed those of GCDC in the plasma. Nevertheless, the more hydrophobic GCDC is transported by NTCP with a significantly greater intrinsic clearance compared to TC [38]. Under specific circumstances, the drug-mediated inhibition of NTCP may protect against cholestasis; for example, bosentan, a dual inhibitor of BSEP and NTCP, was cholestatic in humans but non-cholestatic in rats due to the significantly more potent inhibition of rat Ntcp compared to human NTCP [39].

Of note, NTCP interacts with the scaffolding protein four-and-a-half LIM-domain 2 (FHL2) [40]. The expression of FHL2 suppresses NTCP activity, and FHL2 deficiency aggravates cholestasis in mice.

#### 2.1.2. OATP1B1/OATP1B3

OATP1B1 and OATP1B3 are influx transporters localized in the sinusoidal membrane of hepatocytes. Unlike NTCP, OATP1B1 and OATP1B3 are broad substrate specificity transporters with many drug substrates [41], which implies that drugs inhibiting these transporters are quite common, too.

OATP1B1 is the main transporter mediating sodium-independent BS uptake [42,43], but OATP1B3 and its splice variant OATP1B3-1B7 [44] have also been shown to transport BA/BS [45]. The BS transport profiles of OATP1B1 and OATP1B3 have only recently been characterized [43,45,46,47]. All studies have confirmed the efficient transport of conjugated BA; additionally, OATP1B1 displayed particularly high intrinsic clearance for sulfated BA/BS derivatives. These findings support the use of sulfated and/or glucuronidated BS as biomarkers of the genetic or chemical inhibition of OATP1B1 and OATP1B3 [45,48,49] and, given the increased production of sulfated BA/BS in cholestasis [50], underline the importance of OATP1B1 and OATP1B3 in BA/BS clearance under cholestatic conditions.

Although reports about the effect of *SLCO1B1* polymorphisms on fasting total BA plasma levels have been contradictory [51,52], elevated unconjugated BA levels were observed in *Slco1a/1b* knockout mice [53]. Follow-up studies utilizing knockout models and Ntcp inhibition using Myrcludex showed a more significant role of Ntcp in the hepatic uptake of conjugated BS [54].

#### 2.1.3. BSEP

BSEP plays a pivotal role in the canalicular transport of BA/BS [25]. In humans, the impairment of the gene encoding BSEP, *ABCB11*, causes PFIC-II [55]. Interestingly, BSEP knockout mice display a mild hypercholanemia only, instead of the severe phenotype observed in humans [56]. This is due to compensatory changes such as the increased production of tetrahydroxy BA and upregulation of alternative transport pathways mediated by P-gp (Mdr1a/b) and Mrp4. Bsep/Mdr1a/Mdr1b triple knockouts showed a more severe phenotype [57].

The chemical inhibition of BSEP leads to decreased canalicular efflux and accumulation of BS in the hepatocyte [58]. Unlike non-hepatotoxic BS, hepatotoxic BS damage the inner mitochondrial membrane and precipitate mitochondrial permeability transition [59]. This effect, however, may be experimental system-dependent as isolated mitochondria and HepG2 cells but not HepaRG cells show the direct mitochondrial toxicity of bile salts [60].

#### 2.1.4. MRP2/ABCC2

In hepatocytes, MRP2 is expressed in the canalicular membrane. MRP2/Mrp2 has been linked to cholestasis as a reduction in bile flow was seen in rats upon loss-of-function mutations or the chemical inhibition of Mrp2 [61,62]. However, serum bile acid concentrations do not increase in patients with Dubin–Johnson syndrome, a hereditary MRP2 deficiency [63,64]. A reduction in BS-independent bile flow due to decreased MRP2/Mrp2 function may partly be attributed to the impaired secretion of glutathione (GSH), an MRP2/Mrp2 substrate that is also co-transported with other substrates [65,66,67]. MRP2 has been shown to transport some monovalent bile salts such as glycocholate (GC) and tauroursodeoxycholate but not taurocholate [68,69]. Rat Mrp2 has been shown to transport TC, albeit at a low rate, as well as GC and 3-sulfo-taurolithocholate (3S-TLC) [70].

#### 2.1.5. MRP3/ABCC3

MRP3 is a major efflux transporter expressed on the sinusoidal membrane of hepatocytes [71]. It is generally considered a relief transporter that effluxes endobiotics and xenobiotics into blood [66]. In rats, Mrp3 expression in the liver is increased upon bile duct ligation [72], and Mrp3 expression has been shown to correlate with the sinusoidal efflux of taurocholate [73]. The role of Mrp3 in BA/BS transport in mice is controversial as plasma BA levels did not decrease in a statistically significant manner in *Abcc3* knockout mice [74,75]. Liver BA levels, however, increased by about 50% in *Abcc3* knockouts [75].

In patients with obstructive cholestasis due to gallstones, MRP3 expression was increased 3.4-fold and 4.6-fold at the mRNA and protein levels, respectively [76], which implies an important role for MRP3 in protection against BA/BS overload. Both rat Mrp3 and human MRP3 have been shown to transport BA: whereas GC and 3S-TLC were identified as common substrates, TC was transported by rat Mrp3 only [77,78]. A detailed profiling and kinetic characterization of BA/BS transport by human MRP3 has recently been published [79].

#### 2.1.6. MRP4/ABCC4

MRP4 is expressed on the sinusoidal membrane of hepatocytes [80]. MRP4 has been linked to hepatic bile salt transport as Mrp4 knockout mice showed an impaired cytoprotective response in obstructive cholestasis [81], and Mrp4 was significantly upregulated in Bsep knockout mice [82]. The upregulation of MRP4 was also shown in the inherited BSEP deficiency, PFIC-2 [83], as well as in patients with primary biliary cirrhosis (PBC) and obstructive cholestasis [84]. The bile salt transport profile of human MRP4 was studied in V79 cells overexpressing MRP4 and was shown to be dependent on GSH cotransport [80]. Subsequent substrate specificity studies have shown the transport of cholate and several monovalent BS [85]. In a more recent study using vesicles derived from HEK293 cells overexpressing MRP4 or MRP3, the latter transported BS with significantly higher efficiency [79].

#### 2.1.7. OSTα/β/SLC51A/B

OSTα/β is a complex that functions solely in a heterodimeric form [86]. It is broadly expressed in the body, especially in enterocytes, hepatocytes, and cholangiocytes [87]; therefore, it plays a major role in the enterohepatic and cholehepatic shunts of BA/BS [88,89]. Albeit OSTα/β is a bidirectional facilitative transporter, it is generally considered to provide an efflux function in hepatocytes. It is highly induced in chenodeoxycholate-treated sandwich-cultured human hepatocytes [88,90], and OSTβ (*SLC51B*) deficiency presents with features of cholestasis [91].

In mice, however, Ostα/β may also mediate the uptake of BA as knocking out Ostα provided protection in the obstructed liver. It appears that in the Ostα knockout mice, the compensatory upregulation of the Phase II enzymes (Ugt1a1 and Sult2a1) and basolateral efflux pumps Mrp3 and Mrp4 led to increased efflux and a subsequent increase in the renal elimination of the Phase II metabolites of BA/BS, thus lowering the hepatic BA/BS load [92]. The redundancy and intricate regulation of the basolateral efflux pathways shown in this study clearly demonstrate the importance of the basolateral efflux of BA/BS.

### 2.2. Phospholipid Translocases

A delicate balance of BA/BS, phospholipids, and cholesterol is key to the physicochemical stability of bile. The impaired homeostasis of phospholipids interferes with the excretion of BS and thus precipitates cholestasis. An important cooperation between the two major phospholipid translocases has been shown in a study where overexpression of MDR3 was toxic to HEK293T cells, and this toxicity was counteracted by the concomitant overexpression of the ATP8B1–CDC50A complex [93]. In ATP8A1 knockout mice, but not ATP8A1/MDR3 double knockouts, the BS-induced extraction of phospholipids and proteins from the canalicular membrane was observed [93].

#### 2.2.1. MDR3/ABCB4

The multidrug resistance protein 3 (MDR3) is a phosphatidylcholine floppase. The carriers of functionally defective variants of MDR3 have altered phosphatidylcholine/BS ratios and may develop cholelithiasis, intrahepatic cholestasis in pregnancy, or PFIC-3 [94]. MDR3 has been shown to transport drugs [95]; this capability may explain its broad inhibitor specificity [96].

#### 2.2.2. ATP8B1

ATP8B1 translocates phosphatidylserine from the outer leaflet to the inner leaflet. Consistent with its function as an aminophospholipid flippase, the treatment of Atp8b1-deficient rat hepatocytes with CDC resulted in focal canalicular membrane disruption and the luminal accumulation of NBD-phosphatidylserine [97]. An impaired ATP8B1 function in humans can cause PFIC-1, although in a recent genotype–phenotype correlation study, the severity of genotype was not found to be associated with disease history or prognosis [98]. Van Wessel et al. [98] suggested that the lack of association could be partly due to the indirect role of ATP8B1 in BS transport. It is also possible that mutations in proteins that mediate the trafficking of ATP8B1, but not mutations in ATP8B1 itself, explain the functional impairment observed [99]. Interestingly, to date, there are no known drug inhibitors of ATP8B1 [96].

### 2.3. Regulation of Bile Salt Transport in Hepatocytes

Hepatocytes maintain BA homeostasis by regulating the expression of rate-limiting enzymes that mediate BA synthesis and/or modulating the levels of BA transporters [100]. An abundance of BA is a major regulatory signal. Bile acids are agonists of the farnesoid X receptor (FXR), and intestinal BA reabsorption induces FXR-mediated expression and the subsequent secretion of FGF19, the human ortholog of rodent Fgf15, in the gut. The transcriptional effects of FGF19 are mediated by small heterodimer partners (SHPs).

Acting through FGF4R located in the sinusoidal membrane of hepatocytes, FGF19 represses the expression of CYP7A1, the rate-limiting enzyme of BA synthesis [101]. FXR is expressed in hepatocytes, too, and BA abundance suppresses both the de novo synthesis of BA via repression of CYP7A1 [102] and the uptake of BA/BS via the repression of NTCP and OATPs [103,104]. FXR also facilitates the removal of BA/BS from hepatocytes via the induction of the canalicular BS transporters BSEP [105] and MRP2 [106] as well as the induction of sinusoidal OSTα/β but not MRP4 [107]. Notably, lithocholic acid, the most toxic BA is an FXR antagonist and thus inhibits the removal of BS from hepatocytes [108]. The activity of FXR is regulated partly by SIRT1 (Sirtuin-1). SIRT1 potentiates the transcriptional activity of FXR at multiple levels partly by the deacetylation of FXR [109,110].

The other key regulatory nuclear hormone receptor is the pregnane X receptor (PXR) which induces the expression of sulfotransferase SULT2A1 which, in turn, extensively sulfates BA/BS [50]. PXR agonists also induce the expression of MRP2 [111] and MRP3 [112], and both are shown to transport sulfated BA/BS [77,113] and probably induce MRP4, too [114], which has also been suggested to transport sulfated BA [115].

It has been proposed that BA accumulation triggers adaptive and adverse responses [116]. Adaptive responses include (i) shifting the BA pool towards more soluble BA, such as conjugated BS over unconjugated BA, cholates over chenodeoxycholates, tauro-conjugated BS over glyco-conjugated BS, and sulfated BS, (ii) the suppression of BS uptake, and (iii) the induction of BS efflux [17,37,50]. Therefore, drugs which interfere with adaptive responses might be especially harmful.

Cholestatic, but not non-cholestatic, drugs have recently been shown to inhibit the amidation and sulfation of BA in HepaRG cells [117]. A 24 h treatment with the cholestatic drug cyclosporine A correlated with up to 84% inhibition of the expression of the conjugating enzyme CoA:amino acid N-acyltransferase (BAAT) [118]. The internalization of BSEP is another mechanism that decreases the canalicular clearance of BA/BS [119]. Although mechanistic studies of internalization have been published, drug-induced internalization has not been extensively studied.

### 2.4. Mechanism of Hepatotoxicity of Bile Acids and Bile Salts

Bile acid toxicity affects multiple organelles including the mitochondria, the endoplasmic reticulum (ER), and endosomes/lysosomes. Oxidative stress is one of the adverse responses inflicted on hepatocytes upon BA accumulation, and mitochondria are a major source of oxygen radicals [120]. Unlike non-hepatotoxic BS, hepatotoxic BS damage the inner mitochondrial membrane and precipitate mitochondrial permeability transition (MPT) [59]. BA/BS-induced apoptosis in rat hepatocytes was inhibited by MPT blockers and antioxidants [121]. Whereas it is generally accepted that hydrophobic BS elicit mitochondrial toxicity, the effect may depend on the choice of experimental system. The direct mitochondrial toxicity of BS has been observed in isolated human and rat liver mitochondria as well as HepG2 cells but not HepaRG cells [60,120,122,123]. In addition, in rat liver mitochondria UDC, TUDC and GUDC prevented MPT as assessed via monitoring mitochondrial swelling [122], whereas in HepG2 cells, TUDC failed to prevent CDC-induced apoptosis, and UDC even potentiated CDC-induced necrotic cell death [123]. This apparent discrepancy may partly be accounted for by inter-species differences, considering that CDC is the most abundant bile acid in humans but a minor component in rats [124].

Endoplasmic reticulum (ER) stress is also accompanied by the generation of reactive oxygen species (ROS) because the accumulation of misfolded proteins in the ER leads to the leakage of Ca^2+^ which triggers ROS production [125,126]. Impaired lysosomal function upon accumulation of BA/BS has also been described. BS have been shown to impair autophagosome formation, and the BS-induced blockade of autophagy is mediated by FXR [127]. This may seem counterintuitive as FXR is generally thought to orchestrate a protective response, and autophagy is considered as cytoprotective. A potential interpretation is that excessive autophagy may be detrimental [128], and the effect of FXR is bimodal. This, however, needs to be experimentally confirmed. In addition to the cell-autonomous toxic effects, the overproduction of BA/BS at a tissue level triggers inflammation, with significant contributions from cholangiocytes and the immune cells of the liver [129].

## 3. In Vitro Test Systems for the Study of Bile Acid/Bile Salt Transport

Whereas native BA/BS are broadly considered the most relevant probes to be used in vitro, fluorescent substrates, BA analogs, and unrelated compounds alike have been employed in both vesicular and cellular assays [130,131,132].

The BA/BS uptake by NTCP, OATP1B1, and OATP1B3 has been studied routinely in cell lines overexpressing the respective recombinant uptake transporter [38,43]. Other options include using suspension hepatocytes, adherent hepatocyte cultures, or sandwich-cultured hepatocytes [133,134]. Early tests with OSTα/β were carried out in oocytes [135]. An MDCKII-based double transfectant system co-expressing ASBT and OSTα/β was used in a large inhibition screen of 1280 FDA-approved drugs [136]. More recent studies of OSTα/β inhibition have employed Flp-In-HEK293-based and MDCKII-based transfectants [89,137].

For efflux transporter studies, membrane vesicle assays are the method of choice. Vesicular transport studies on bile salt transport by all major transporters including BSEP, MRP2, MRP3, and MRP4 have been published [77,79,85,113,138,139]. Sandwich-cultured hepatocytes have also been used to investigate bile salt efflux [140]. Liver spheroids were found similarly suitable for the assessment of BA/BS transporter function using fluorescent BA as substrates and were leveraged to model drug-induced cholestasis [141,142]. Thanks to the disparate substrate recognition of BSEP and MRP2, the canalicular BSEP function can be studied in these holistic cellular models with reasonable specificity. In contrast, the basolateral efflux functions provided by MRP3, MRP4, and OSTα/β are difficult to differentiate unless using knockout or knockdown models.

### Testing of Bile Salt Transport to Assess Cholestatic Liver Injury

It is generally accepted that interference with BS transport is a key causal factor in cholestasis. Major publications from both the industry and the International Transporter Consortium have recommended the testing of BSEP inhibition. The relevant papers with suggested cut-off values [143,144,145,146,147,148,149] are summarized in Table 1. Most studies employ a vesicular transport assay with taurocholate as a probe, albeit some cholestatic compounds have been shown to act via repression of BSEP expression, which can only be investigated in cellular models [150].

Studies referring to C_ss_/IC_50_ values use 0.05 and 0.1 as cut-offs [143,144,145], whereas IC_50_/C_hep,inlet,u_ < 26 (ca. C_hep,inlet,u_/IC_50_ > 0.04) may also serve as an indicator [146]. For compounds in the discovery phase where the C_plasma_ values are still only predicted and may thus be unreliable, risk assessment can be based solely on IC_50_, and different groups have suggested cut-off values of 25 [145] and 50 µM [147]. Aleo et al. have applied a complex scoring system, with the highest score (most severe category) assigned to compounds with IC_50_/C_max,total_ ≤ 1 and the safe zone for BSEP inhibition starting at IC_50_/C_max,total_ > 100 [148]. In other studies, the utility of in vitro BSEP inhibition for predicting DILI has been confirmed without recommending a specific cut-off [96,108].

Whether in vitro inhibition assays for other bile acid and bile salt transporters may add value to DILI risk assessment is a matter of debate. Although the overall effect of the inhibition of influx and efflux transporters is challenging to quantify, initial integrative models have been published [151,152]. Inhibition of any of the MRPs (i.e., MRP2, MRP3, or MRP4) with a cut-off of C_ss_/IC_50_ ≥ 0.1 has been considered a significant contributor to cholestasis and was suggested to be included in decision-making [144]. Inhibition of the sinusoidal efflux functions MRP3, MRP4, and OSTα/β has also been acknowledged as significant in several studies [153,154]. Conversely, the inclusion of MRP2, MRP3, and MRP4 inhibition did not improve the predictability of DILI in a more recent study [149]. Meanwhile, the ITC paper recommended a broader panel that included OATP1B1, OATP1B3, and NTCP in addition to MRP2-4 as a follow-up to clinically relevant (C_ss_/IC_50_ ≥ 0.1) BSEP inhibition [145].

The genetic impairment of MDR3 function leads to PFIC-3, and the inhibition of MDR3 by antifungals has been linked to cholestasis [155,156]. The possible cholestatic effect of MDR3 inhibition was addressed in a large study that assayed 125 drugs on hepatocytes using d9-phosphatidyl-choline as a probe [148]. “Most-DILI concern compounds” inhibited MDR3 and BSEP with IC_50_ < 20 µM. However, whether the substrate transport was specific to MDR3 remained a concern [145], and testing the inhibition of transporters other than BSEP was found to have minimal beneficial impacts on the prediction of DILI [148].

Drugs may not only contribute to DILI by inhibiting bile salt efflux. Other mechanisms that may precipitate or aggravate hepatotoxicity include the blockade of FXR-mediated adaptive responses [157,158] or the inhibition of amidation and sulfation [117]. Hence, the mechanism of DILI is complex, and even the clinical presentation of cholestasis may be highly variable [17,159]. Other than bile salt transport inhibition, new testing paradigms encompass the mitochondrial toxicity, generation of ROS, cytotoxicity that may be related to the accumulation of bile salts, and formation of reactive metabolites. High doses (≥100 mg), lipophilicity (cLogP ≥ 3), and high molecular weights (>600) are further attributes that have been linked to hepatotoxic potential [17,147,148].

## 4. Conclusions

For compounds in the discovery stage, a BSEP IC_50_ ≤ 25 µM threshold has been suggested [145]. In the presence of additional compound- and dose-related risk factors, such as MW > 600, cLogP > 3, and oral dose >100 mg, BSEP IC_50_ ≤ 50 µM may be a reasonable cut-off [147,148]. For compounds in the discovery/clinical Phases I–II with predicted total C_ss,plasma_/BSEP IC_50_ ≥ 0.1, testing for the inhibition of other relevant hepatocyte bile acid transporters may be needed [145].

## Figures and Tables

**Table 1:** Recommendations for the in vitro evaluation of cholestatic risk. Abbreviations: BEI, biliary excretion index; hMPCC, hepatocyte micropatterned co-culture; SCH, sandwich-cultured hepatocytes; TC, taurocholate; VT, vesicular transport.

Source	Morgan et al., 2013 [144]	Schadt et al., 2015 [143]	Riede et al., 2017 [146]	Yucha et al., 2017 [147]	Kenna et al., 2018 [145]	Aleo et al., 2020 [148]	Hafey et al., 2020 [149]
**BSEP assay method**	VT (probe: TC)	VT (probe: TC)	VT (probe: TC)	VT (probe: TC)	VT (probe: TC) or SCH (probe: TC)	VT (probe: TC)	VT (probe: TC)
**Recommended DILI risk cut-off/score for BSEP**	C_ss_/IC_50_ ≥ 0.1	C_plasma_/IC_50_ > 0.05	IC_50_/C_hep,inlet,u_ < 26: cholestasis common; 26 < IC_50_/C_hep,inlet,u_ < 529: cholestasis rare	IC_50_ < 50 µM	Drugs in discovery: IC_50_ ≤ 25µM Drugs in discovery to Phase I–II: C_ss_/IC_50_ ≥ 0.1	IC_50_/C_max,total_: Score = 1 if >50–≤100); Score = 2 if >10–≤50); Score = 3 if >1–≤10); Score = 4 if ≤1);	IC_50_ < 5 µM
**Recommendations for other transporter interaction assays**	MRP2, MRP3, and MRP4 recommended				MRP2, MRP3, MRP4, OSTα/β, NTCP, OATP1B1, and OATP1B3 recommended in the case of clinically relevant BSEP inhibition		MRP2, MRP3, and MRP4 *not* recommended
**Other recommended assays**		Reactive metabolites; mitochondrial toxicity; cellular toxicity	In vitro K_p,uu_		Reactive metabolites; mitochondrial toxicity; oxidative stress	Cytotoxicity; mitochondrial dysfunction (isolated mitochondria); mitochondrial dysfunction (cell)	Inhibition of TC transport in hMPCC: IC_50_ for clearance and BEI
**Further considerations**				MW > 600; cLogP > 3; dose > 100 mg/day		Physicochemical properties	

## Data Availability

Not applicable.
